# Effect and Mechanisms of State Boredom on Consumers’ Livestreaming Addiction

**DOI:** 10.3389/fpsyg.2022.826121

**Published:** 2022-04-05

**Authors:** Nan Zhang, Jian Li

**Affiliations:** ^1^School of Economics and Management, Beijing Jiaotong University, Beijing, China; ^2^School of Management, Minzu University of China, Beijing, China

**Keywords:** state boredom, livestreaming addiction, life meaning perception, sensation seeking, China consumers

## Abstract

With the rapid development of livestreaming marketing in China, consumers spend an increasing amount of time watching and purchasing on the platform, which shows a trend of livestreaming addiction. In the early stage of the COVID-19 pandemic, the addiction exacerbated by a surge of boredom caused by home quarantine. Based on the observation of this phenomenon, this research focused on whether state boredom could facilitate consumers’ livestreaming addiction and explored the associated mechanisms of this relationship. Based on three studies, this research found that state boredom had a positive effect on consumers’ livestreaming addiction, and this relationship worked through the mediating effect of consumers’ sensation seeking. We further verified a moderated mediation effect of consumers’ life meaning perception, where the indirect effect of state boredom on consumers’ livestreaming addiction *via* consumers’ sensation seeking existed for high and low levels of life meaning perception, but in opposite directions. The conclusions provided theoretical and practical implications of livestreaming marketing and healthy leisure consumption.

## Introduction

The livestreaming industry is growing rapidly worldwide. Livestreaming platforms are recognized as a unique form of social media, which extend the forms of computer-mediated communications from text and image to audio and video. As a special combination of multiple media forms, livestreaming allows individuals to publicly broadcast live video streams, chat with broadcasters and other users, purchase products, and send gifts at the same time (Hamilton et al., 2014). The livestreaming platform includes all kinds of live streams ranging from eating, gaming, singing, shopping, to traveling anytime and anywhere. It offers a new approach for individuals to relax and communicate by an exciting and more interactive social media.

With the rapid development of the livestreaming industry, individuals become addicted to livestreaming platforms and apps. Livestreaming addiction is defined as the consumers watching for a long time and purchasing frequently on the livestreaming platform. This phenomenon has become more serious during the COVID-19 pandemic. The COVID-19 pandemic restricted people’s activities, and related leisure consumption suffered, such as traveling, eating at restaurant, outdoor activities, etc. The perceived severity of COVID-19 led to an increase in boredom and sensation-seeking expression, which significantly promoted consumers’ post-pandemic consumption willingness (Deng et al., 2020). According to IIMedia Research, China’s livestreaming e-commerce industry has grown rapidly when consumers were isolated at home because of the COVID-19 pandemic. For example, in the first quarter of 2020, 41.7% of users of livestreaming e-commerce shopped 5–8 times per week on average, 37.5% of users shopped 1–4 times, 6.7% of users shopped 9–12 times, 3.3% of users shopped 13 or 16 times, and 0.8% of users shopped more than 16 times per week on average.

Researchers have paid sufficient attention to users’ addiction or continuance intention on other types of social media, such as gaming disorders (Jeon et al., 2019), computer addiction (Cho and Kim, 2010), Internet addiction (Kuss and Lopez-Fernandez, 2016), social media addiction (Brand et al., 2019), and addiction to heavy viewing (Horvath, 2004). However, extant literature still lacks a comprehensive explanation for livestreaming addiction. Limited studies have tried to figure out this question from two aspects: on the one hand, the factors of individual traits, such as escape from loneliness (Chen and Chang, 2019), information seeking (Hilvert-Bruce et al., 2018), flow experience (Chen and Lin, 2018), perceived value (Singh et al., 2021), and Big Five personality traits (Cheng et al., 2019); on the other hand, a social relationship between broadcasters and audiences, such as the parasocial interaction between broadcasters and audiences (Lim et al., 2020), the social identification of the co-viewers, and the sense of community within the streaming room (Lim et al., 2012; Hu et al., 2017).

Although some factors for livestreaming addiction have been recognized, few researchers have examined the relationship between state boredom and livestreaming addiction. Boredom refers to a negative experience of desiring but being unable to engage with the environment or in satisfying activities (Biolcati et al., 2018). According to the stability across time and situations, boredom can be categorized into proneness boredom and state boredom. Proneness boredom refers to the stable tendency of boredom generated by individuals in various situations. It is a part of personality traits, related to self-adjusting ability, intrinsic motivation, and values (Musharbash, 2007). On the contrary, state boredom is a temporary boring experience generated by an individual in a specific situation. It is a subjective feeling that can be triggered by monotonous and repetitive external stimuli. In recent years, scholars realize that proneness boredom is not enough to understand boredom itself, but a deeper understanding of boredom and its influence on individual decisions and behaviors could be possible by exploring state boredom (Bench and Lench, 2019). However, previous studies mainly discussed proneness boredom and its effects on some addictive behaviors, such as mobile phone addiction (Chou et al., 2018; Wang et al., 2020), Internet addiction (Lin et al., 2009), and Facebook addiction (Donati et al., 2022), and less is talked about state boredom. Therefore, paying attention to the effect of state boredom on addiction is a supplement to boredom and addiction theoretical research.

The mechanisms of state boredom on livestreaming addition may be related to sensation seeking and life-meaning perception. Sensation seeking is defined as “a trait by the seeking of varied, novel, complex, and intense sensations and experiences and the willingness to take physical, social, legal, and financial risks for the sake of such experience” (Zuckerman, 1996). Scholars believe that boredom is characterized by a lack of interest, concentration, and motivation (Huang et al., 2010). Additionally, the self-regulatory process helps bored people to seek sensation (Reisenzein, 2017), and sensation seeking is positively related to Internet addiction (Müller et al., 2016). Therefore, we proposed the mediating effect of sensation seeking on the relationship between state boredom and livestreaming addiction.

Life meaning is defined as a generally stable sense of purpose in life and an accompanying sense of fulfillment (Baumeister, 1991), and a contributor to psychological health (Brassai et al., 2011). Faced with a disaster context, individuals with high-perceived life meaning could cope well and be satisfied with life (Drescher et al., 2012). However, the role of life meaning in boredom has rarely been studied. In addition, life meaning could prevent individuals’ unhealthy behaviors, such as Internet addiction (Zhang et al., 2015). Therefore, life meaning may modify the positive effect of state boredom on livestreaming addiction.

Specifically, this research intends to address the following research questions. Could state boredom lead to livestreaming addiction, just like proneness boredom? How does state boredom promote livestreaming addiction? In addition, under what condition, the positive effect of state boredom on livestreaming addiction would be weakened? Based on the theoretical analysis and practical observation, this study proposed that state boredom has a positive effect on livestreaming addiction, and this effect works through the mediating effect of sensation seeking. The boundary effect of the above relationship is the perception of life meaning.

The structure of this research proceeds as follows: Section Theoretical Background and Research Hypotheses reviewed the theoretical background of state boredom and livestreaming addiction and proposed three hypotheses. Section Materials and Methods described the research method and showed the results to test the three hypotheses. Section Conclusion and Implications discussed the conclusion, theoretical and practical implications, research limitations, and future research directions.

## Theoretical Background and Research Hypotheses

### State Boredom and Livestreaming Addiction

In clinical psychology, boredom is often defined as an emotional state characterized by unpleasant feelings, a lack of stimulation, and low physical arousal (Harris, 2000). Individuals describe boredom as stress, anxiety, exhaustion, pain, and suffering (Mann and Robinson, 2009). Other symptoms include feeling that time is passing so slowly, escaping out of boredom through physical and mental relief (e.g., daydreaming), and talking in a slow and monotonous way (Biolcati et al., 2018). Therefore, scholars believe that boredom is a state of under-stimulation, under-arousal, and a lack of psychological participation associated with dissatisfaction and that individuals try to cope with it by seeking extra stimulation (Brissett and Snow, 1993).

To get rid of boredom, individuals indulge themselves in behaviors full of stimulants, such as overeating, gambling, television, and Internet addiction (Musharbash, 2007). Previous studies have shown that boredom is positively correlated with addictive behaviors such as alcoholism, drug abuse, Internet addiction, and gambling (Pekrun et al., 2010). In terms of food consumption, boredom highly correlated with consumers’ overeating (Wilson, 1986), significantly increased the frequency of eating (Robin, 1975), led to eating disorders (Stickney and Miltenberger, 1999), and enabled obese people to eat more food than normal-weight people faced with boring tasks (Abramson and Stinson, 1977). For social media addiction, Whelan et al. (2020) suggested a strong association between proneness boredom and both information and communication overload, which in turn increased social media fatigue.

Boredom is recognized as one of the common causes of addiction, such as Internet addiction (Chou et al., 2018), Facebook use (Donati et al., 2022), gambling behavior (Mercer and Eastwood, 2010), and smartphone addiction (Wang et al., 2020). Boredom is a negative state of under-arousal, and people usually try to escape from boredom and achieve physiological arousal through media, which leads to media dependence (Khang et al., 2013). Livestreaming is a combination of novel and exciting activities, such as chatting, gambling, viewing videos, shopping, and traveling. Thus, these activities make live streaming a good tool for people with state boredom to easily arouse their psychology, which in turn reinforces livestreaming addiction.

State boredom is a temporary boring experience generated by an individual in a specific situation. Previous studies on boredom focused more on proneness boredom, but in fact, state boredom is more common in our daily life, such as college students’ sense of meaninglessness and boredom in study and life (Nett et al., 2011) and white-collar workers’ state boredom in the bottleneck period of work (Loukidou et al., 2009). When individuals are under daily state boredom, they may indulge in the popular livestreaming platforms. As a new form of social media, the characteristics of livestreaming provide individuals with the opportunities of seeking entertainment and killing time. In a relaxing atmosphere, individuals can interact with the broadcasters and participate in a lucky draw, which has resulted in livestreaming addiction, especially when individuals are boring. Therefore, we suggested the first hypothesis:

H1: State boredom promotes individuals’ livestreaming addiction.

### Mediating Effect of Sensation Seeking

State boredom is positively related to sensation seeking. Boredom is caused by monotony, which means that there are no external stimuli or a single stimulus (Hill and Perkins, 1985). People feel bored because they lack interest or stimulation caused by the surrounding environment (Sansone et al., 1992). The self-regulatory process triggered by state boredom can influence specific behaviors by sensation seeking (Van Tilburg and Igou, 2012), which is designed to make the boring situation more interesting or challenging (Jessi et al., 2009). Individuals who suffer from boredom would actively seek out more and stronger complex external stimuli (Reisenzein, 2017). Bryant and Zillmann (1984) found that subjects in the state boredom group chose more exciting television programs than those in the state stress group. Moynihan et al. (2015) found that state boredom increased a person’s preference for stimulus-inducing food (such as candy) but not for non-stimulus-inducing food (such as saltines). At the same time, sometimes people choose negative stimulation to relieve state boredom. Wilson et al. (2014) left the participants alone in a room for 15 min to induce their state boredom. They were then told that they were free to choose whether or not to receive an electric shock during the solitude. It was found that individuals would rather receive a small negative electric shock than keep waiting.

Sensation seeking leads to addictive behaviors. Numerous studies have been conducted to explain why consumers use social media addictively. Some studies found that seeking entertainment and killing time are the strong predictors of social media overload (Quan-Haase and Young, 2010; Ku et al., 2013). Meanwhile, sensation seeking has proved to trigger addictive behaviors in previous research, such as Internet addiction (Müller et al., 2016). Livestreaming addiction could be seen as one kind of Internet addiction. Besides, as one of the most effective ways to relax and entertain, livestreaming can make boring people feel new, exciting, and fun. Therefore, when consumers are bored, they may frequently watch livestreaming and buy products to seek stimulation. Based on this, we proposed the second hypothesis, the mechanisms, and the mediating effect of sensation seeking:

H2: Sensation seeking mediated the positive influence of state boredom on consumers’ livestreaming addiction.

### Moderated Mediation Effect of Life Meaning Perception

State boredom is closely related to life meaning. A central feature of boredom is a lack of perceived meaning (Fahlman et al., 2009). Boredom indicates that the current situation is purposeless, which is a meaningful threat (Van Tilburg and Igou, 2012). Researchers suggest that the loss or failure of developing meaning in life is a critical factor for boredom (Van Tilburg et al., 2018).

Life meaning could decrease the negative results of boredom. For example, studies proved that life meaning prevents Internet addiction (Zhang et al., 2015). This is because life meaning is associated with feelings of control (Martela and Steger, 2016), and those with self-control or high self-esteem demonstrated lower tendency to be addicted (Leung, 2007). Therefore, life meaning might affect how people cope with boredom, and whether they would stave off boredom through livestreaming addiction.

Recent research indicated that boring people attempted to escape from the meaninglessness associated with boredom by engaging in simulating activities (Moynihan et al., 2017). The sensations involved in these simulating acts and addiction may help to distract people from meaninglessness (Moynihan et al., 2015). Therefore, when boring people try to seek sensation, if they have high life-meaning perception, the meaninglessness threat signaled by state boredom is low and then may weaken the positive effect of state boredom on sensation seeking and addiction; and if they have low life-meaning perception, the meaninglessness threat signaled by state boredom is high, they have a strong motivation for sensation seeking and addiction, and the positive effect of state boredom on sensation seeking and addiction is enhanced. In a word, life meaning moderated the relationship of state boredom on consumers’ sensation seeking and addiction.

In general, individuals suffering from state boredom tend to engage in activities that help restore a sense of meaning (Barbalet, 1999). Life meaning is proved to modify the association of state boredom on media use, which is served as a risk factor for the negative psychological outcomes when individuals experienced boredom during the COVID-19 outbreak in China (Miao et al., 2020). Therefore, when individuals have a high sense of life meaning, they will regulate their behaviors to maintain a high sense of life meaning, to reduce sensation seeking and addictive behavior in a state of boredom. However, when the sense of life meaning is low, they will have lower limits on sensation seeking and addictive behaviors in a state of boredom, thus promoting consumers to engage in more addictive behaviors. Based on the above analysis, hypothesis 3 is proposed.

H3: Life-meaning perception has a mediating moderation effect on the relationship between state boredom and livestreaming addiction. When the perception of life meaning is low, state boredom will increase livestreaming addiction through sensation seeking; when the perception of life meaning is high, state boredom will reduce livestreaming addiction through sensation seeking.

**FIGURE 1:**
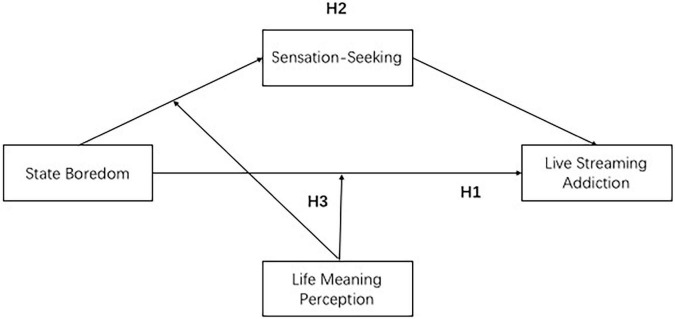
Research framework.

Based on the above hypotheses, we explored the effect of state boredom on livestreaming addiction and the mediating role of sensation seeking and the moderating role that life meaning played on the mediating relationship. The overall research framework is shown in Figure 1.

## Materials and Methods

Totally, three studies were conducted to test the above hypotheses. Study 1 was a self-reported survey to test the main effect of state boredom and livestreaming addiction. Study 2 was a survey-embedded randomized experiment to test the mediation effects of sensation seeking on the relationship of state boredom and livestreaming addiction, and Study 3 was a self-reported survey to test the moderated mediation effect of life meaning perception on the relationship among state boredom, sensation seeking, and livestreaming addiction.

Participants were recruited from the online survey platform Credamo.^1^ At the beginning of the survey and experiment, all participants signed informed consent online. The participants were guaranteed anonymity and allowed to discontinue the survey at any time. They were told that the survey was a sociological study that consists of several unrelated sub-surveys. The survey included an attention check test, which needs to be answered carefully, and the corresponding reward can only be obtained after passing the researcher’s review. Each participant can get 10 Yuan as a reward.

### Study 1

#### Participants

A number of 100 participants were recruited using the sample database on the Credamo. Among them, 34% are men, 69% aged 21–30 years, and 31% aged 31–40 years (refer to Table 1 for more information of demographics). The participants have rich experience with livestreaming. Totally, 65% of participants indicated that livestreaming shopping is the most popular type of livestreaming. On average, they spent 1,165.8 Yuan [standard deviation (SD) = 1,053.785] in the last month on the livestreaming platform and watched livestreaming for 355.09 min (SD = 255.916) per week.

**TABLE 1:** Description of participants’ demographics in Study 1.

Education	*N*	Percentage
Secondary education	1	1%
Associate’s degree	12	12%
Bachelor’s degree	74	74%
Master’s degree	13	13%
**Occupation**		
Students	7	7%
State-owned enterprises	23	23%
Private enterprises	56	56%
Foreign-invested enterprises	4	4%
Public institutions	9	9%
Civil servant	1	1%
**Household income monthly (RMB)**		
<5,000	2	2%
5,000–9,999	14	14%
10,000–14,999	24	24%
15,000–19,999	22	22%
20,000–24,999	16	16%
25,000–29,999	10	10%
≥30000	12	12%
**Perceived SES**		
Between the bottom and the middle of SES	23	23%
The middle of SES	63	63%
Between the middle and the upper of SES	14	14%

*N = 100.*

#### Procedures and Measures

Study 1 was a survey, to test the main effect of state boredom on consumers’ livestreaming addition. To guarantee the reliability and validity of the questionnaires, we choose the mature scales that have been verified with high reliability and validity. The measurement was originally in English and was subsequently translated into Chinese, following the back-translation process (Brislin, 1970).

After obtaining informed consent, participants were asked to answer the scales of state boredom, their daily behaviors on the livestreaming platform, livestreaming addition, current affects, and finally the demographics. After the data were qualified, the reward was paid.

First, state boredom was measured using a 29-item state boredom scale adapted from Fahlman et al. (2011), which had good reliability (α = 0.965). Participants were asked to indicate the degree of boredom on a 7-point Likert scale (1 = strongly disagree and 7 = strongly agree). The sample items included “I am stuck in a situation that I feel is irrelevant,” “Everything seems repetitive and routine to me,” “I feel empty,” and so on. Second, livestreaming addiction was measured, adapted from the Bergen Facebook Addiction Scale (Andreassen et al., 2012), with 18 items (α = 0.933). Participants were asked how often have they “Spent a lot of time thinking about livestreaming or planned use of livestreaming?” “Thought about how you could free more time to spend on livestreaming?” and so on. Each item was scored on a five-point scale (1 = very rarely, 2 = rarely, 3 = sometimes, 4 = often, and 5 = very often). Third, the positive and negative affects were tested, because the research showed that the boredom would have an effect on person affects (Alda et al., 2015). We chose five related affects from the Positive and Negative Affect Schedule (PANAS; Watson et al., 1988), namely, excited, irritated, bored, impatient, and happy, with a 5-point Likert scale (1 = very slightly, 5 = very strongly). Fourth, demographic information was collected, such as gender, age, education, household income, and perceived socioeconomic status.

#### Results

Given the nature of the single-shot cross-sectional survey, we checked whether there is a common method bias before the formal data analysis. Harman’s one-factor analysis was conducted following the recommendations of Podsakoff and Organ (1986). An exploratory factor analysis using a maximum likelihood solution was conducted on all of the items of key variables in this study. A number of seven factors emerged with eigenvalues larger than 1.00, which suggests that more than one factor underlies the data. Moreover, the first factor accounted for only 42.596% of the total variance, which suggests that the common method variance may not be a serious concern in this study (Eby and Dobbins, 1997).

##### Description of State Boredom *and Livestreaming Addiction*

Factor analysis was conducted for state boredom and livestreaming addiction. State boredom was proved to combine as one factor (KMO = 0.934, *p* < 0.000) and also for livestreaming addiction (KMO = 0.899, *p* < 0.000). Besides, we conducted a confirmatory factor analysis (CFA) for livestreaming addiction, and the resulting 18-item scale showed an excellent fit to the data (NFI = 0.923, CFI = 0.987, RMSEA = 0.043), averaged the items, and got the mean-centered score of state boredom and livestreaming addiction. Means, SD, and correlation are shown in Table 2.

**TABLE 2:** Means, standard deviations (SDs), and correlations of variables in Study 1.

	M	SD	Correlation
1 State boredom	2.39	1.05	
2 Livestreaming addiction	2.69	0.75	0.576**

**p < 0.01.

##### Main Effect of State Boredom on Livestreaming Addiction

Using SPSS 26.0, we regressed consumers’ state boredom on their livestreaming addiction, respectively, in three models to test H1. In model 1, state boredom was the only predictor of livestreaming addiction. In model 2, demographic variables were included, and in model 3, five affects were analyzed as the control variables. As expected, state boredom had a significantly positive effect on consumers’ livestreaming addiction (β = 0.576, *t* = 6.981, *p* < 0.000). The result was consistently significant when controlling for the demographics in model 2 (β = 0.636, *t* = 7.031, *p* < 0.000) and for the PANAS in model 3 (β = 0.592, *t* = 5.101, *p* < 0.000), and H1 was approved. Detailed information is shown in Table 3.

**TABLE 3:** The effect of state boredom on livestreaming addiction.

	Model 1	Model 2	Model 3
**Main effect**			
State boredom	0.576***	0.636***	0.592***
**Controls**			
Gender		–0.110	–0.088
Age		–0.016	0.016
Education		0.066	0.028
Work		0.191	0.089
Household income monthly		0.133	0.136
Perceived SES		–0.140	–0.209
Excited			0.250
Irritated			0.076
Bored			0.189
Impatient			0.060
Happy			0.166
R^2^	0.332	0.391	0.490
ΔR^2^	0.325	0.345	0.419

N = 100; ***p < 0.001.

### Study 2

In Study 1, we tested the main effect of state boredom on livestreaming addiction based on the participants’ self-reported survey. In Study 2, two more things were conducted. One was the manipulation of state boredom. State boredom was primed with a typical psychological experiment, a one-factor between-subject design, namely, state boredom (yes vs. no). The other was the inclusion of sensation seeking to test whether sensation seeking played a mediating role in the relationship between state boredom and livestreaming addiction.

#### Participants

A total of 100 participants were recruited from the sample database on the Credamo platform, who have not joined in Study 1. They were divided into two groups: the boring group (*N* = 50) and the non-boring group (*N* = 50). Among them, 36 are men (36%), and their average age was 28.51 (SD = 5.76, min = 19, max = 56). All the participants had experience in watching or purchasing in livestreaming (refer to Table 4 for information on other demographic variables).

**TABLE 4:** Description of participants demographics in Study 2.

Education	*N*	Percentage
Secondary education	1	0.8%
Associate’s degree	16	13.3%
Bachelor’s degree	88	73.3%
Master’s degree	15	12.5%
**Occupation**		
Student	8	6.7%
State-owned enterprises	27	22.5%
Private enterprises	69	57.5%
Foreign-invested enterprises	5	4.2%
Public institutions	9	7.5%
Civil servant	2	1.7%
**Household income monthly (RMB)**		
<5,000	2	1.7%
5,000–9,999	17	14.2%
10,000–14,999	25	20.8%
15000–19,999	26	21.7%
20,000–24,999	22	18.3%
25,000–29,999	12	10%
≥30,000	16	13.3%
**Perceived SES**		
Between the bottom and the middle of SES	26	21.7%
The middle of SES	78	65%
Between the middle and the upper of SES	16	13.3%

*N = 100.*

#### Procedures and Measures

Before the formal experiment, participants were asked to give informed consent online, just like Study 1. The whole procedures of the formal experiment included four steps. When the data were reviewed and qualified, the reward was paid. First was the manipulation of state boredom. Participants were randomly assigned into two conditions: the boring condition and the non-boring condition. They were asked to recall a time in their life when they felt bored or non-bored and to describe it in as much detail as possible. Second was the manipulation check. Participants answered two questions. “How boring do you think it is today?” (1 = not boring at all, 7 = extremely boring) and “Do you often feel bored in the last week?” (1 = very rarely, 7 = very often). Third was the measurement of the addiction scale (Andreassen et al., 2012), which had good reliability (α = 0.916). Fourth was the measurement of the Impulsive Sensation Seeking [ImpSS; Zuckerman et al. (1993)] scale. This scale included 19 questions (α = 0.862): seven questions were about impulsivity and 12 questions were about sensation seeking. Every question was scored for 1 (yes) or 0 (no), and the final score of the impulsivity and sensation seeking is the sum of all questions. A higher score indicated a higher level of impulsivity and sensation seeking. Finally, demographic information, such as gender, age, education, work, and household income, was collected.

#### Results

##### Manipulation Check of State Boredom

The results showed that there is a significant difference in the perception of boredom between the boring group and the non-boring group, and people in the boring group perceived more state boredom (M_*boring*_ = 4.02, SD_*boring*_ = 1.68) than those in the non-boring group [(M_*notboring*_ = 2.62, SD_*notboring*_ = 1.31), *F*_(1_,_99)_ = 21.557, *p* < 0.000]. There is also a significant difference in the frequency of boredom between the boring group and the non-boring group, [*F*_(1,99)_ = 17.490, *p* < 0.000, M_*boring*_ = 4.08, SD_*boring*_ = 1.76, M_*notboring*_ = 2.68, SD_*notboring*_ = 1.58]. The result showed that the manipulation of state boredom is effective.

##### Effect of State Boredom on Livestreaming Addiction

Factor analysis was conducted on the livestreaming addiction scale and was combined into one factor, KMO = 0.883, *p* < 0.000, and it averaged the 18 items and got the mean-centered score of livestreaming addiction, M = 2.92, SD = 0.67. Using SPSS 26.0, ANOVA was conducted to test the main effect of consumers’ state boredom on their livestreaming addiction. Results showed a significant difference in livestreaming addiction between the two conditions [*F*_(1,99)_ = 4.292, *p* = 0.041]. The addictive behavior of participants in the boring condition (M_*boring*_ = 3.05, SD_*boring*_ = 0.70) was significantly higher than that in the non-boring group (M_*notboring*_ = 2.78, SD_*notboring*_ = 0.62). Thus, H1 was approved again.

##### Mediating Effect of Sensation Seeking

Factor analysis was conducted on the ImpSS scale of the livestreaming platform and was combined into one factor, KMO = 0.743, *p* < 0.000, it averaged the 19 items and got the mean-centered score of livestreaming addiction, *M* = 0.33, SD = 0.22. Following model 4 of the PROCESS Macro (Hayes, 2012), we performed a 5,000-resampling bootstrapping-moderated mediation analysis with state boredom as the independent variable (0 = boring condition, 1 = non-boring condition), sensation seeking as the mediator, consumer’s livestreaming addiction as the dependent variable, and demographics as the control variables. The result showed that impulsive sensation seeking played a complete mediating role and that state boredom promotes consumers’ livestreaming addiction, β = −0.1059, 95% CI (−0.2488, −0.0207) (refer to Table 5 for more information). Besides, we conducted a regression analysis according to the mediation analysis of Baron and Kenny (1986), and the results are shown in Table 6. Therefore, H2 was supported.

**TABLE 5:** Mediating effects of sensation seeking by bootstrapping analysis.

Impulsivity and sensation seeking	Effect	BootSE	BootLLCI	BootULCI
Indirect effect	–0.1059	0.0577	–0.2488	–0.0207
Direct effect	–0.1686	0.1370	–0.4405	0.1034
Total effect	–0.2744	0.1325	–0.5373	–0.0116

**TABLE 6:** Mediating effects of sensation seeking by regression analysis.

	Model 1		Model 2		Model 3	
Variables	Livestreaming		Livestreaming		Sensation	
	addiction		addiction		seeking	
	β	*p*	β	*p*	β	*p*
State boredom	−0.205	0.041	−0.126	0.222	−0.327	0.001
Sensation-seeking			0.242	0.020		
F	4.292		5.035		11.745	
R^2^	0.042		0.094		0.107	
ΔR^2^	0.032		0.075		0.098	

Using behavioral experiment in Study 2, we manipulated state boredom in different conditions and the result verified H1, that is, state boredom would increase consumers’ livestreaming addictive behaviors. H2 was proved, that is, consumers’ sensation seeking motivation played a mediating role in the relationship between state boredom and consumers’ livestreaming addiction.

Studies 1 and 2 measured and primed state boredom with different methods. We found that state boredom significantly promoted consumers’ livestreaming addictive behaviors, which means that consumers are more likely to indulge in livestreaming and purchasing behavior under the condition of state boredom. At the same time, we found that consumers’ sensation seeking played a mediating role in the above relationship. The boundary of mediating effect of sensation seeking is discussed in Study 3. In other words, under what circumstances will state boredom be restricted to improve consumers’ livestreaming addictive behavior through sensation seeking will be discussed in that study.

### Study 3

Study 3 aimed to examine the moderating effect of life meaning on the mediating effect of sensation seeking on the relationship of state boredom and consumers’ livestreaming addiction. In Study 3, measurement of life meaning was added, and a new and simple sensation seeking scale was used. In Study 2, the ImpSS scale included seven items for impulsivity, which were not the key variable we cared about. In view of our interest in distinguishing between impulsivity and sensation seeking, we changed to use only the six Zuckerman items that clearly index thrill or novelty seeking (Steinberg et al., 2008).

#### Participants

A number of 120 subjects were recruited from the sample database on the Credamo platform, which excluded people who participated in Studies 1 and 2. Among them, 38 are men (31.7%), 81 aged between 21 and 30 years (67.5%), 38 aged between 32 and 40 years (31.7%), and 1 aged between 41 and 50 years (0.8%).

#### Procedures and Measures

Before the formal survey, participants were asked to provide informed consent online, just like Studies 1 and 2. The whole procedures of the formal survey included five steps. When the data were reviewed and qualified, the reward was paid. First was the measurement of state boredom with the same scale as used in Study 1 (Fahlman et al., 2011), which has good reliability (α = 0.965). Second was the measurement of the livestreaming addiction scale (Andreassen et al., 2012) with good reliability (α = 0.931). Third was the measurement of consumers’ sensation seeking (Steinberg et al., 2008) with six items (α = 0.847), which includes “I like to have new and exciting experiences and sensations even if they are a little frightening,” “I like doing things just for the thrill of it,” “I sometimes like to do things that are a little frightening,” “I’ll try anything once,” “I sometimes do ‘crazy’ things just for fun,” and “I like wild and uninhibited parties.” We adopted seven-point Likert scale, with one representing “strongly disagree” and seven representing “strongly agree.” Fourth was the measurement of life-meaning perception [Meaning in Life Questionnaire, MLQ; Steger et al. (2006)] with 10 items (α = 0.808). The typical items included “I understand my life’s meaning,” “My life has a clear sense of purpose,” and participants chose their agreement on a seven-point Likert scale (1 = absolutely untrue, 7 = absolutely true). Finally, demographic information was collected.

#### Results

A common method bias was conducted first, just like that in Study 1. An exploratory factor analysis using a maximum likelihood solution was conducted on all of the items of key variables in this study. Totally, eighteen factors emerged with eigenvalues larger than 1.00, which suggests that more than one factor underlies the data. Moreover, the first factor accounted for only 33.231% of the total variance, which suggests that common method variance may not be a serious concern in this study (Eby and Dobbins, 1997).

##### Description of Key Variables

Factor analysis was conducted for state boredom, livestreaming addiction, sensation seeking, and life meaning. State boredom was proved to combine as one factor (KMO = 0.945, *p* < 0.000) and also for livestreaming addiction (KMO = 0.912, *p* < 0.000), sensation seeking (KMO = 0.847, *p* < 0.000), and life meaning perception (KMO = 0.879, *p* < 0.000). Factor analysis averaged the items and got the mean-centered score of the four key variables. Means, SDs, and correlation are shown in Table 7.

**TABLE 7:** Means, SDs, and correlations of variables in Study 3.

	*M*	SD	Correlation		
			1	2	3
1. State boredom	2.57	1.04			
2. Live-streaming addiction	2.65	0.73	0.574**		
3. Sensation seeking	4.44	1.00	–0.069	0.316**	
4. Life meaning	5.46	0.85	−0.391**	0.077	0.348**

**p < 0.01.

##### Main Effect of State Boredom on Livestreaming Addiction

Using SPSS 26.0, we tested the main effect of consumers’ state boredom on their livestreaming addiction by ANOVA. The results showed a significant difference in the livestreaming addictive behavior between the two conditions, *F*_(1,118)_ = 13.158, *p* < 0.000. The addiction of participants in the boring condition (M_*boring*_ = 2.88, SD_*boring*_ = 0.70) was significantly higher than that in the non-boring condition (M_*notboring*_ = 2.42, SD_*notboring*_ = 0.70). Thus, H1 was approved again.

##### Moderated Mediation Effect of Life Meaning and Sensation Seeking

Following model 8 of the PROCESS Macro (Hayes, 2012), we performed a 5,000-resampling bootstrapping-moderated mediation analysis with state boredom as the independent variable, life meaning as the moderator, sensation seeking as the mediator, and live-streaming addiction as the dependent variable. The result showed a moderated mediation effect: life meaning perception moderated the mediation effect of sensation seeking between state boredom and livestreaming addiction [indirect effect = −0.0653, 95% CI = (−0.1329, −0.0229)]. In particular, when the life meaning was low, the indirect effect of state boredom on livestreaming addiction through sensation seeking was significantly positive [0.0315, 95% CI = (0.0038, 0.0802)]. In contrast, when the life meaning was high, the indirect effect of state boredom on livestreaming addiction through sensation seeking was significantly negative [−0.0797, 95% CI = (−0.1709, −0.0234)].

The results indicated that the effect of state boredom on consumers livestreaming addiction *via* sensation seeking existed in both high and low levels of life meaning perception, but in opposite directions. When life meaning perception was low, state boredom would increase livestreaming addiction through sensation seeking; however, when the life meaning perception was high, state boredom would decrease livestreaming addiction through sensation seeking. Therefore, H1, H2, and H3 were approved.

## Conclusion and Implications

### General Discussion

This article investigated the relationship between state boredom and livestreaming addiction and the mechanisms of the moderated mediation of sensation seeking and life meaning perception. Three studies were conducted to test the main effect of state boredom on consumers’ live-streaming addiction, the mediating effect of consumers’ sensation seeking, and the moderated mediation effect of life meaning perception. The results indicated that state boredom, one common kind of boredom, could lead to livestreaming addiction, and when individuals are under state boredom, they are motived by sensation seeking to have addictive behaviors on the livestreaming platforms. When consumers possess high life meaning perception, the state boredom will reduce sensation seeking and further reduce the livestreaming addictive behavior. However, when the perception of life meaning is low, bored consumers can hardly control their own behavior, which will strengthen the promotion effect of sensation seeking on livestreaming addiction.

The finding of state boredom and sensation seeking has been proved in the previous research. For example, Deng et al. (2020) showed that boredom from limited activities during the COVID-19 pandemic has positive effect on individuals’ sensation seeking expressions. Bench and Lench (2019) manipulated boredom in high and low conditions and found that boredom is a seeking state and boredom prompts the pursuit of novel (even negative) experiences. The boredom in both of the above studies is actually state boredom, and the results mean that state boredom promotes sensation seeking.

The positive effects of state boredom and sensation seeking on individuals’ addiction is consistent with previous research. Wang et al. (2020) found that both proneness boredom and sensation seeking could promote smartphone addiction. However, they did not investigate the relationship between boredom and sensation seeking, in which sensation seeking was an independent variable, rather than a mediator of the relationship between boredom and addition.

This research argued that life meaning perception could moderate the mediating effect of sensation seeking on the positive effect of state boredom on livestreaming addiction. If ones’ life meaning perception is high, he could control the sensation seeking expression and then decrease the desire of livestreaming addiction. In the previous literature, meaning threat involves a strong self-regulatory process and helps individuals to change the state of boredom (Leary et al., 1986; Van Tilburg and Igou, 2011). For example, boredom increases people’s evaluation of inner group and also demeaning of external group, so as to establish their sense of meaning (Van Tilburg and Igou, 2011). Similarly, people sometimes get nostalgic and indulge in reveries to offset the lack of meaning caused by boredom (Van Tilburg et al., 2013). However, this research paid attention to life meaning perception, rather than the meaning threat and meaning seeking, to explain the mechanism of state boredom on livestreaming addiction.

### Contributions and Implications

This research had both theoretical contributions and practical implications. Theoretically speaking, first, this research extended the literature of boredom and consumer’s behaviors. Previous research focused on proneness boredom and its effects on consumer’s behaviors [e.g., Chou et al. (2018) and Wang et al. (2020)]. However, this research figured out that state boredom, which could be stimulated by environment and happen on everyone, could also influence consumers’ behaviors. We explored how state boredom leads to livestreaming addiction and thus further expands research on the mechanism of addictive behaviors in the field of consumer’s behavior. Second, this research supplied the existing literature about social media addiction. Social media addiction has been studied about Facebook addiction (Ryan et al., 2014), addiction to social network sites (Griffiths et al., 2014), Twitter addiction (Saaid et al., 2014), and microblogging dependence (Wang et al., 2015). We paid attention to the topic of livestreaming addiction, a new kind of social media addiction, and explored the mechanisms of it.

In practice, this research can help to guide consumers to correctly deal with the negative impact of state boredom, can reduce the consumption caused by livestreaming addiction, and can promote health livestreaming time consumption. Specifically, on the one hand, marketers or broadcasters can appropriately enhance the interest and attraction of the livestream room and thus increase individuals’ watching time and shopping amount in the livestreaming platform by stimulating consumers’ sensation seeking. On the other hand, for consumers, high perception of life meaning can be used to inhibit the promoting effect of state boredom on livestreaming addiction. Therefore, individuals could think about their life meaning when they are watching live streaming to help allocate their leisure time rationally and form healthy livestreaming consumption habits.

### Limitation and Future Research

There are two deficiencies in this research, which can be made up in future. First, individuals’ livestreaming addiction was measured through self-report questionnaire and individuals’ viewing data were lacking. Considering the reality that individuals tend to watch livestreaming at night after work, this real situation is difficult to be designed and present in the laboratory. In the future, two sources of data could be used to analyze the livestreaming addiction behaviors: company-shared data of individuals’ real behaviors on the livestreaming platform or some instruments can be used to record individuals’ livestreaming behaviors at their leisure boredom time. Second, considering the limitation of data collected online, we adopted the situation recall method to deal with the priming of state boredom. Although manipulation was verified successfully, the method was relatively simple. In future, classical initiation methods of state boredom can be adopted and manipulated in the laboratory, which include repetitive action tasks (Markey et al., 2014), cognitive tasks (Van Tilburg and Igou, 2011), and video tasks (Merrifield and Danckert, 2014). Besides, we could provide some real livestreaming video materials to test the participants’ addiction intention and real watching time. The video materials could include different kinds of products, such as cultural product and food, and entertainment products (e.g., singing, dancing, and traveling).

## Data Availability Statement

The datasets generated during and/or analyzed during the current study are available from the corresponding author on reasonable request.

## Ethics Statement

Ethical review and approval was not required for the study on human participants in accordance with the local legislation and institutional requirements. Electronic written informed consent for participation was required for this study in accordance with the national legislation and the institutional requirements.

## Author Contributions

NZ developed the theoretical framework and worked on data analysis and manuscript writing. JL worked on literature review and manuscript writing. Both authors contributed to the article and approved the submitted version.

## Conflict of Interest

The authors declare that the research was conducted in the absence of any commercial or financial relationships that could be construed as a potential conflict of interest.

## Publisher’s Note

All claims expressed in this article are solely those of the authors and do not necessarily represent those of their affiliated organizations, or those of the publisher, the editors and the reviewers. Any product that may be evaluated in this article, or claim that may be made by its manufacturer, is not guaranteed or endorsed by the publisher.

## Footnotes

1
https://www.credamo.com/#/
